# Reciprocal Interactions between Nematodes and Their Microbial Environments

**DOI:** 10.3389/fcimb.2017.00144

**Published:** 2017-04-27

**Authors:** Ankur Midha, Josephine Schlosser, Susanne Hartmann

**Affiliations:** Department of Veterinary Medicine, Institute of Immunology, Freie Universität BerlinBerlin, Germany

**Keywords:** nematode, helminth, microbiota, antimicrobial peptides, antibiotic resistance

## Abstract

Parasitic nematode infections are widespread in nature, affecting humans as well as wild, companion, and livestock animals. Most parasitic nematodes inhabit the intestines of their hosts living in close contact with the intestinal microbiota. Many species also have tissue migratory life stages in the absence of severe systemic inflammation of the host. Despite the close coexistence of helminths with numerous microbes, little is known concerning these interactions. While the environmental niche is considerably different, the free-living nematode *Caenorhabditis elegans* (*C. elegans*) is also found amongst a diverse microbiota, albeit on decaying organic matter. As a very well characterized model organism that has been intensively studied for several decades, *C. elegans* interactions with bacteria are much more deeply understood than those of their parasitic counterparts. The enormous breadth of understanding achieved by the *C. elegans* research community continues to inform many aspects of nematode parasitology. Here, we summarize what is known regarding parasitic nematode-bacterial interactions while comparing and contrasting this with information from work in *C. elegans*. This review highlights findings concerning responses to bacterial stimuli, antimicrobial peptides, and the reciprocal influences between nematodes and their environmental bacteria. Furthermore, the microbiota of nematodes as well as alterations in the intestinal microbiota of mammalian hosts by helminth infections are discussed.

## Introduction

Parasitic nematodes are responsible for widespread morbidity in humans and animals. It is estimated that approximately 1.5 billion people are infected with one or more of these organisms (Hotez et al., 2008; World Health Organization, 2017) which also pose a considerable burden for animal production (Eijck and Borgsteede, 2005; Nganga et al., 2008). The extraordinary success of these parasites speaks to their ability to withstand a multitude of stresses such as host immune pressures and infectious challenges from microbes. Most parasitic nematodes inhabit the intestines of their hosts, co-existing with numerous microbial species. In studying these dynamics, researchers have focused extensively on the host-parasite relationship; more recently, the role of the microbiota as a major third party in the relationship is better appreciated due to diverse and far-reaching influences in health and disease (Donaldson et al., 2016). Much attention in these studies is given to host immune mechanisms while the interactions between nematodes and their microbial environments are largely overlooked. Due to many technical and biological challenges associated with studying parasites, these questions can be very difficult to address. The free-living nematode *Caenorhabditis elegans* (*C. elegans*) is frequently found in nature amongst a diverse microbiota on decaying organic matter (Frézal and Félix, 2015). *C. elegans* has been very well characterized and its interactions with bacteria studied in considerable detail. As such, these findings might be conveyed to parasitic nematodes and greatly inform our understanding of how parasites interact with the host-microbiota, as many immune-related pathways and responses may be conserved (Tarr, 2012; Rosso et al., 2013). This review will summarize current knowledge regarding how parasitic nematodes and their microbial environments may influence each other, supplemented with insights from work in *C. elegans*.

## Nematodes and their microbial environments

Studies on intestinal nematodes report a range of alterations to the composition of the microbiota, ranging from increased or decreased diversity, dysbiosis, or in some cases no identifiably significant changes (summarized in Tables 1, 2). Responses by *C. elegans* to numerous microbes have also been studied. Accordingly, parasitic helminths illuminate our understanding of how microbial communities can be altered by the presence of nematodes whereas studies of *C. elegans* can explain how microbial populations impact nematode physiology, reproduction and growth; considered together, one can better understand how these multifactorial biological systems operate. Herein, we focus most of our discussion on selected members of the genera *Trichuris* and *Ascaris*, as well as the rodent parasite *Heligmosomoides polygyrus bakeri* and the free-living *C. elegans*. These organisms have been selected due to their importance to human and animal health along with the abundance of available data in the literature, though additional studies from other helminths are considered where appropriate.

**Table 1:** **Effects of helminth infection on host microbiota and metabolism in humans, macaques, and pigs**.

**Host and helminth(s) species**	**Microbiota diversity**	**Microbiota composition**	**Changes in host metabolism**	**Sample site(s)**	**Population details**	**References**
**HUMAN**						
*T. trichiura*	→	n.r.	n.r.	Feces	Rural Ecuadorians	Cooper et al., 2013
	↓	*Prevotella* ↑	n.r.	Feces	Rural Malaysians	Ramanan et al., 2016
*A. lumbricoides, T. trichiura* and hookworm (mixed infections)	↓	*Streptococcus* ↑	n.r.	Feces	Rural Ecuadorians *A. lumbricoides* and *T. trichiura*	Cooper et al., 2013
	↑	Prevotellaceae, Mollicutes, Bacteroidales Alphaproteobacteria ↑	Carbohydrate metabolism ↓	Feces	Rural Malaysians *A. lumbricoides, T. trichiura*, and hookworm	Lee et al., 2014
*N. americanus*	→	n.r.	n.r.	Feces	Healthy volunteers (gluten-free diet; 8 wpi)	Cantacessi et al., 2014
	↑	Bacteroidetes and Bacteroidia ↑; *Lachnospira, Ruminococcus*, Firmicutes, Tenericutes ↓	Gluten tolerance ↑	Feces	Volunteers with celiac disease (8 wpi), gluten administration in parallel	Giacomin et al., 2015
	n.r.	n.r.	SCFA ↑	Feces	Healthy volunteers (8 wpi)	Zaiss et al., 2015
**MACAQUE**						
*T. trichiura*	↑	Cyanobacteria ↓; Tenericutes and Bacteroidetes ↑; bacterial attachment to mucosa ↓	n.r.	Colon	Chronic helminth infection in a colitis model	Broadhurst et al., 2012
**PIG**						
*T. suis*	→	*Ruminococcus, Oscillibacter* and *Succinivibrio*↓; *Mucispirillum* and *Paraprevotella* ↑	Altered fatty acid metabolism and carbohydrate metabolism, amino acid availability↓	Colon	Larval stage infection (21 dpi)	Li et al., 2012
	n.r.	*Ruminococcus Oscillibacter* and *Succinivibrio* ↓; *Campylobacter* ↑	n.r.	Colon	Chronic infection (53 dpi)	Wu et al., 2012
*A. suum*	↓	*Prevotella* ↑	SCFA ↑	Colon	54 dpi	Paerewijck et al., 2015

*→, no change; ↑, increase; ↓, decrease; n.r., not reported; SCFA, short chain fatty acids; dpi, days post-infection; wpi, weeks post-infection; Nod2, nucleotide-binding oligomerization domain-containing protein 2; WT, wild type*.

**Table 2:** **Effects of helminth infection on host microbiota and metabolism in mice**.

**Host and helminth(s) species**	**Microbiota diversity**	**Microbiota composition**	**Changes in host metabolism**	**Sample site(s)**	**Infection phase**	**References**
***MUS MUSCULUS***						
*T. muris*	↓	Bacteroidetes ↓; *Mucispirillum, Bifidobacterium*, Lactobacillaceae, Proteobacteria and Firmicutes ↑	n.r.	Cecum Colon Feces	Chronic infection	Holm et al., 2015
	↓	*Parabaceroides, Prevotella* and Bacteroidetes ↓; *Mucispirillum* and Rikenellaceae ↑	weight gain and carbohydrate metabolism ↓	Feces	Chronic infection	Houlden et al., 2015
	↑*Nod2* deficient mice; ↓ in WT mice	*B. vulgatus, Prevotella* and *Bacteroides* ↓; Lachnospiraceae, Lactobacillales and Clostridiales ↑	n.r.	Feces	Acute infection (21 dpi)	Ramanan et al., 2016
*H. p. bakeri*	n.r.	Lactobacillaceae, Clostridiaceae, Ruminococcaceae and Lachnospiraceae ↑	n.r.	Ileum Cecum	Acute infection (14 dpi)	Walk et al., 2010
	n.r.	*Baceroides, Clostridium, Lactobacillus* and Enterobacteriaceae ↑	n.r.	Ileum Cecum Colon	Acute infection (14 dpi)	Rausch et al., 2013
	n.r.	Enterobacteriaceae and Lactobacillaceae ↑	n.r.	Duodenum Feces	28 dpi	Reynolds et al., 2014
	n.r.	Clostridiales ↑	SCFA ↑	Cecum	28 - 42 dpi	Zaiss et al., 2015
*N. brasiliensis*	→	Firmicutes and SFB ↓; Bacteroidetes and Actinobacteria ↑	n.r.	Jejunum Ileum Cecum Colon Feces	11 dpi	Fricke et al., 2015
***APODEMUS FLAVICOLLIS***						
*H. p. bakeri, Syphacia*. *Hymenolepsis* and *M. muris* (mixed infections)	→	Firmicutes/Bacteroidetes ratio ↑ (with *H. p. bakeri*)	carbohydrate metabolism ↑ (with *H. p. bakeri*)	stomach small intestine cecum colon	Wild caught mice, likely chronic exposure to helminths	Kreisinger et al., 2015

*→, no change; ↑, increase; ↓, decrease; n.r., not reported; SCFA, short chain fatty acids; dpi, days post-infection; wpi, weeks post-infection; Nod2, nucleotide-binding oligomerization domain-containing protein 2; WT, wild type; SFB, segmented filamentous bacteria*.

### The nematodes

#### Trichuris

The whipworms *Trichuris trichiura, T. suis*, and *T. muris* infect humans, pigs, and mice respectively. The life cycles of these species are comparable and infection begins with ingestion of developed eggs (Bethony et al., 2006; Klementowicz et al., 2012). Hatching occurs within hours of ingestion, liberating L1 larvae which invade the intestinal wall and undergo successive molts to the L4 stage by 3 weeks post-infection (pi) and finally develop into mature adults by 12 weeks pi (Bethony et al., 2006). Trichurids inhabit the most dense and diverse microbial environments of their hosts: the cecum and colon (Klementowicz et al., 2012). They can survive here for 1–2 years with individual females laying up to 5,000 eggs per day (Bethony et al., 2006).

#### Ascaris

The roundworms *A. lumbricoides* and the closely related *A. suum* infect humans and pigs respectively. As with Trichuriasis, Ascariasis also spreads via the fecal-oral route in humans as well as in pigs (Dold and Holland, 2011). Within 3 h, ingested eggs containing L3 larvae hatch and by 18 h pi the larvae begin their tissue migratory phase, passing through the liver after invading the cecum and proximal colon (Murrell et al., 1997). The larvae reach the lungs and pharynx by days six to eight pi (Roepstorff et al., 1997), are swallowed and can then be found in the small intestine as L4 stage larvae before further developing into mature adults. Adult Ascarids reside in the small intestine for around 1 year with individual females producing hundreds of thousands of eggs per day (Dold and Holland, 2011).

#### H. p. bakeri

The trichostrongyloid *H. p. bakeri* is amongst the most common helminth parasites of rodents and a well characterized model for chronic intestinal nematode infection (Wu et al., 2012). Infection is initiated by ingestion of L3 larvae which migrate into the mucosa of the small intestine. By day three pi the larvae develop into L4 within the mucosa before returning to the lumen of the duodenum by day nine pi where they develop into, and remain as, egg-laying adults for approximately 12 weeks in the wood mouse (*Apodemus sylvaticus*) and more than twice as long in BALB/c laboratory mice (Robinson et al., 1989; Gregory et al., 1990; Behnke et al., 2009).

#### C. elegans

*C. elegans* is a free-living, bacterivorous nematode found predominantly in humid temperate areas (Félix and Duveau, 2012; Frézal and Félix, 2015). These nematodes can be identified in feeding and reproductive stages in rotting fruit and herbaceous stems. The life cycle is characterized by freshly hatched L1 stage larvae undergoing multiple molts before reaching adulthood in as little as 3 days. Under stressful conditions including crowding, limited food, and heat stress, L1 individuals will pursue an alternate life cycle through a pre-dauer L2 (L2d) stage, followed by a non-feeding, stress-resistant alternate L3 stage called “dauer.” Dauer individuals can survive for several months without food before reentering a relatively normal development cycle when conditions are more favorable. In the laboratory, the reference N2 strain is typically cultured on a diet of the rather innocuous *Escherichia coli* OP50. In contrast to controlled laboratory conditions, *C. elegans* shares its natural habitat with a variety of organisms, including bacteria, phages, fungi, isopods, arthropods, and other nematodes (Félix and Duveau, 2012). Additionally, worms can be found in states of starvation or constipation in their natural environments (Barrière and Félix, 2005).

### Parasitic nematodes influence the host-intestinal microbiota

**Figure 1:**
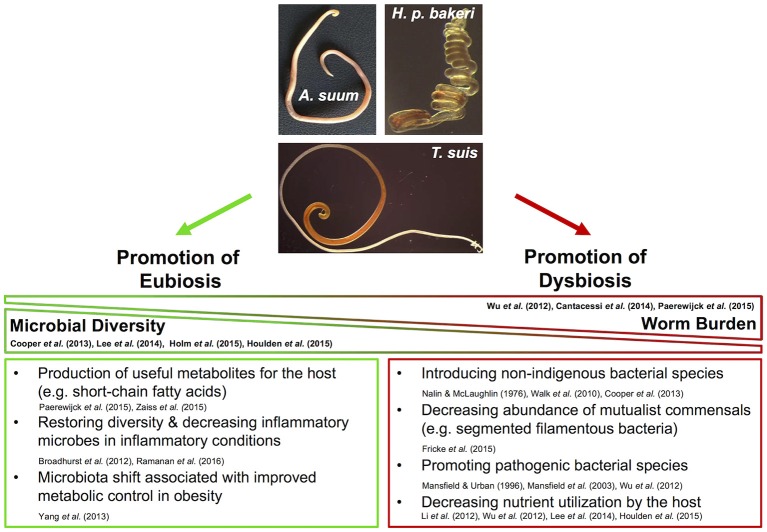
**Diverse influences of nematodes on the gut microbiota of their hosts**.

The mammalian intestine is home to approximately 3.8 × 10^13^ microbes from all three domains of life, archaea, bacteria, and eukaryotes, collectively referred to as the microbiota (Sender et al., 2016). The different regions of the intestinal tract form divergent habitats, varying in bacterial type and density. In humans, an estimated 10^3^–10^4^ microbial cells/mL intestinal content reside in the small intestine (Sender et al., 2016). Considerably more bacterial diversity and density is found in the large intestine with as many as 10^11^ cells/mL contents representing thousands of different species (Zoetendal et al., 2008; Sender et al., 2016). Interestingly, microbial density and diversity of intestinal regions are inversely correlated with concentrations of host-derived antimicrobials; specialized secretory Paneth cells are a key source of α-defensins, lysozymes, and C-type lectins and are particularly prominent in the proximal intestine, the intestinal site of lowest microbial richness (Bevins and Salzman, 2011; Donaldson et al., 2016). Additionally, persistence of common gut commensals is partially mediated by resistance to host defense molecules (Cullen et al., 2015). Whether these factors influence helminth niche selection remains to be determined. Various conditions such as age, diet, health status and genetic background can impact the host microbiota composition. Maturation of the gut microbiota is characterized by increasing diversification with age while age-related changes are also due to exposure to a more varied diet (David et al., 2014; Odamaki et al., 2016). Analysis of human fecal samples can detect rapid, diet-mediated changes in microbial composition. For example, consumption of animal products increases the abundance of bile-tolerant species and decreases abundance of species that metabolize plant-derived polysaccharides (David et al., 2014). Dysbiosis, a potentially pathogenic imbalance of microbial communities, can be precipitated by exposure to pharmaceutical substances such as antimicrobials as well as the presence of infectious agents (Donaldson et al., 2016). Acute antibiotic treatment can drastically perturb the gut microbiota, decreasing species diversity (Dethlefsen et al., 2008). Alarmingly, while some changes appear reversible, other alterations can be detected in fecal samples even years after a short course of antibiotic treatment (Jernberg et al., 2007). A major clinical implication of a disturbed gut microbiota is elevated risk of enteric infection, such as with *Clostridium difficile*, which itself is associated with decreased microbial diversity (Milani et al., 2016). Many studies have now shown that intestinal nematodes influence their microbial niches as they establish themselves as part of their wider environment within the host; therefore, the role of helminths in dysbiosis is an area of active investigation (Figure 1).

The gut microbiota of humans, pigs, and mice is dominated by two of the 29 known bacterial phyla: Bacteroidetes and Firmicutes, with lower abundance phyla differing between hosts and including Actinobacteria, Deferribacteres, Proteobacteria, Spirochaetes, Tenericutes, and Verrucomicrobia (Leser et al., 2002; Eckburg et al., 2005; Consortium, 2012; Nguyen et al., 2015; Weldon et al., 2015). The diversity of the microbiota is thought to reflect the health of the intestine, with far reaching implications for the overall health of the host; greater species diversity contributes to healthy metabolic and immune functioning. Dysbiosis is associated with a reduction in intestinal biodiversity, predisposing to the outgrowth of particularly harmful bacterial species (Carding et al., 2015). The literature is mixed with respect to whether or not helminths cause dysbiosis, and some reports have indicated beneficial effects in therapeutic settings. The few studies assessing the influence of helminths on intestinal microbial communities are a mix of clinical observations and animal experiments, employing different analytical tools and acquiring microbiota samples from different sources. Hence, it is difficult to draw meaningful and generalizable conclusions from the available evidence. Still, through a careful reading of the literature, some common threads emerge.

Analysis of fecal samples from children in rural Ecuador revealed widespread helminth infection (Cooper et al., 2013). Children co-infected with both *T. trichiura* and *A. lumbricoides* appeared to have a decreased microbial diversity compared to uninfected children and children with *T. trichiura* single infections who did not differ from uninfected individuals. Interestingly, in a subset of children with mixed infections the authors also reported a higher abundance of *Streptococcus* spp., not usually dominant in healthy individuals. Taken together, these data suggest dysbiosis in the presence of *A. lumbricoides*, while *T. trichiura* single infections did not result in drastic alterations to the fecal microbiota. Another indication of dysbiosis associated with *Ascaris* infection comes from a study of *A. suum*-infected pigs which showed worm burden-dependent decreased bacterial diversity compared to control animals (Paerewijck et al., 2015). One study in humans found no difference in community structure of fecal samples in healthy volunteers infected with *Necator americanus* (Cantacessi et al., 2014). In contrast, a study of fecal samples from helminth-infected (*T. trichiura, A. lumbricoides*, hookworm) individuals in rural Malaysia found a positive association between helminth-colonization and microbiota diversity (Lee et al., 2014). Burrowed in the epithelia, *T. trichiura* likely interacts with the mucosal microbiota, the composition of which is known to differ considerably from fecal communities (Eckburg et al., 2005). Combined with the obstacles to sampling the small intestine, the site of *A. lumbricoides* and *N. americanus* infections, it is difficult to draw firm conclusions from these human studies regarding beneficial or harmful effects of helminth infections with respect to intestinal microbial composition. Though in the case of *Ascaris*, the local effects seen in the porcine small intestine (Paerewijck et al., 2015) correspond with the distal effects seen in human feces (Cooper et al., 2013).

Animal studies of helminthiases can offer more depth compared to human studies, accounting for the limitations associated with sampling only the fecal microbiota. During the larval stage, *T. suis* appears not to disrupt the porcine colonic microbiota (Li et al., 2012); however, chronic infections in pigs demonstrate worm burden-dependent disruption (Wu et al., 2012). *T. suis* also appears to promote *Campylobacter* infection in pigs, intensifying colitis disease severity (Mansfield and Urban, 1996; Mansfield et al., 2003; Wu et al., 2012). Chronic *T. muris* infections in mice considerably decrease overall microbial diversity, an effect that appears reversible upon worm clearance (Holm et al., 2015; Houlden et al., 2015). In these studies, the murine microbiota also showed a shift away from Bacteroidetes in favor of Firmicutes (Holm et al., 2015; Houlden et al., 2015). A study of wild mice has also observed an increased Firmicutes/Bacteroidetes ratio in helminth-infected individuals (Kreisinger et al., 2015) whereas mice experimentally infected with the small intestinal nematode *Nippostrongylus brasiliensis* showed a decrease of Firmicutes while increasing Bacteroidetes (Fricke et al., 2015). Though *N. brasiliensis* decreases the Firmicutes/Bacteroidetes ratio, it also promotes the reduction of segmented filamentous bacteria (SFB) which are thought to prevent colonization by bacterial pathogens, as it was shown in SFB-colonized mice with enhanced resistance to the pathogenic *Citrobacter rodentium* (Ivanov et al., 2009; Fricke et al., 2015). Mice with chronic *T. muris* infections (Holm et al., 2015; Houlden et al., 2015) as well as those with acute *H. p. bakeri* infections (Rausch et al., 2013) have higher abundance of Enterobacteriaceae, a family shown to overgrow during intestinal inflammation (Lupp et al., 2007) and strongly correlated with Crohn's disease (Gevers et al., 2014) and *C. difficile* infection (Milani et al., 2016) in humans. These data indicate a propensity for helminths to associate with a simplified microbiota; however, whether these observations are generalizable across helminth infections in diverse mammalian hosts remains to be determined; nonetheless, these animal experiments are suggestive of helminths promoting dysbiosis in their hosts.

Despite the evidence for dysbiosis, a fairly consistent finding across various helminth infections is an increased abundance of Lactobacillaceae (Walk et al., 2010; Rausch et al., 2013; Reynolds et al., 2014; Fricke et al., 2015; Holm et al., 2015; Houlden et al., 2015; Ramanan et al., 2016), a family composed primarily of *Lactobacillus* spp., currently under intense investigation for use as probiotics (Walter, 2008; Salvetti et al., 2012). Additionally, studies employing intestinal nematodes as therapeutic interventions to treat intestinal inflammatory conditions have shown beneficial outcomes (Broadhurst et al., 2012; Giacomin et al., 2015; Ramanan et al., 2016). Macaques with idiopathic chronic diarrhea show signs characteristic of dysbiosis such as decreased diversity, increased bacterial attachment to the intestinal mucosa, and increased abundance of Enterobacteriaceae (Broadhurst et al., 2012). Bacterial attachment and abundance of Enterobacteriaceae were effectively decreased by *T. trichiura* infection, while bacterial diversity was restored, indicating a protective effect of helminth infection in this setting. Similarly, mice deficient in *Nod2* are susceptible to Crohn's disease and can be colonized by inflammatory *Bacteroides* spp., while *T. muris* infection protects against pathogenic colonization (Ramanan et al., 2016). In patients with celiac disease, low-level infection with *N. americanus* increases gluten tolerance (Croese et al., 2015), while also increasing microbial richness when combined with gluten consumption (Giacomin et al., 2015). Taken together, these findings indicate that intestinal nematodes possess great therapeutic potential and may promote microbial restoration in conditions of pre-existing dysbiosis.

From taxonomic data referring to the microbiota composition alone it is challenging to conclude whether intestinal nematodes contribute to or ameliorate dysbiosis. As such, the influence of helminths on gut metabolic profiles may offer more clues. Methodological and technological advances allow for metabolic profiling of gut microbiota, along with identification and quantification of metabolites of interest (Vernocchi et al., 2016). Such experiments can be quite informative, though they are currently far less abundant than those reporting taxonomic changes. Metabolomic analysis of the colonic microbiota of pigs infected with *T. suis* demonstrated reduced capacity to metabolize and utilize carbohydrates relative to control animals, an effect seen in acute and chronic infections (Li et al., 2012; Wu et al., 2012). Interestingly, *T. suis*-infected pigs also showed signs of altered fatty acid metabolism, including higher levels of oleic acid relative to control pigs (Li et al., 2012). Notably, oleic acid possesses antibacterial activity (Dilika et al., 2000) and may therefore influence microbial composition during *Trichuris* infection. Mice chronically infected with *T. muris* also showed signs of reduced carbohydrate and amino acid metabolism and nutrient uptake compared to naïve animals, features which likely contribute to decreased weight gain during helminth infection (Houlden et al., 2015). Observational analysis of helminth-infected humans has attributed decreased carbohydrate metabolism to *Ascaris* infection (Lee et al., 2014). In contrast, wild mice infected with helminths showed mixed effects with respect to metabolomic shifts (Kreisinger et al., 2015). Notably, wild mice with *H. p. bakeri* appeared to have an increased capacity to metabolize carbohydrates; however, metabolites were not measured and mouse weights were not reported. Together, these observations suggest helminth-mediated microbiota changes tend to reduce metabolic capability in the gut, ultimately manifesting as nutritional deficiencies.

While dietary carbohydrate utilization may be impaired by intestinal nematodes, other experiments demonstrate potential benefits of helminth infection such as increased abundance of intestinal short-chain fatty acids (SCFAs), typically produced from microbial processing of indigestible oligosaccharides (Paerewijck et al., 2015; Zaiss et al., 2015; Vernocchi et al., 2016). SCFAs can serve as energy sources and anti-inflammatory compounds and are therefore under investigation for their therapeutic potential (Vernocchi et al., 2016). Increased SCFAs have been detected during infection with *H. p. bakeri* in mice, *A. suum* in pigs, and *N. americanus* in humans (Paerewijck et al., 2015; Zaiss et al., 2015). Furthermore, *H. p. bakeri* and *A. suum* can produce SCFAs directly (Tielens et al., 2010; Zaiss et al., 2015). Reduced weight gain associated with helminth infection may also prove to be therapeutic in different circumstances, as illustrated by Yang et al. in a study of obese mice and mice fed obesity-inducing diets (Yang et al., 2013). In agreement with other observations, *N. brasiliensis* infection attenuated weight gain. Importantly, helminth infection decreased adiposity and hepatic lipid storage while improving glycemic control, implying a reversal of metabolic disease. These studies indicate that microbiota alterations and metabolomic changes associated with intestinal nematodes may offer certain benefits.

The mixed observations described herein highlight the difficulties of generalizing findings from these studies, simultaneously hinting at the massive potential to apply these insights in diverse therapeutic settings. Animal studies designed to induce strong immune responses are typically a result of a high infection dose and thus high worm burdens and may not reflect average worm burdens seen in nature. Indeed, some of the aforementioned animal experiments demonstrate microbiota changes suggestive of dysbiosis (Li et al., 2012; Wu et al., 2012; Holm et al., 2015; Houlden et al., 2015). In contrast, observations from natural settings or studies inducing low-level helminth infections suggest less dramatic changes (Cantacessi et al., 2014; Kreisinger et al., 2015). Additionally, when intestinal nematodes are introduced into individuals with intestinal inflammation or metabolic abnormalities, they appear beneficial (Broadhurst et al., 2012; Yang et al., 2013; Giacomin et al., 2015). Taken together, these data emphasize co-evolution of helminths, gut microbiota, and mammalian hosts, such that helminths can be considered less strictly as intruders but rather members of the gut macrobiota (Gause and Maizels, 2016). In order to establish themselves in the mammalian intestine, rather than being solely beneficial or causing outright harm, nematodes must shape the niche such that a new ecological balance is found. This task is aided by some microbes and challenged by others, and the insights gained from understanding these dynamics can provide opportunities to better understand and perhaps manipulate incredibly complex biological systems. It should be noted that many potential mechanisms have been identified which act in concert to shape the intestinal microbiota. Immune mechanisms such as modifying the balance between Th1/Th17 vs. Th2 responses as well as the induction of regulatory T cells have been characterized and discussed elsewhere (Reynolds et al., 2015; Gause and Maizels, 2016; Zaiss and Harris, 2016). Furthermore, the bulk of work done thus far has focused primarily on bacteria and far less is known about the archaeal and fungal components of intestinal microbial communities. In addition to metabolomic factors touched upon here, important considerations which are relatively under-studied and not frequently discussed are the direct signals between helminths and microbes. These reciprocal interactions are considered below.

### Environmental microbes impact *C. elegans* physiology

The study of intestinal parasites is particularly amenable to understanding alterations to microbial communities of the gut, though the microbial influence on helminths is relatively difficult to assess in these systems. As a model organism with many available genetic tools, *C. elegans* can bridge some of the knowledge gaps encountered when studying parasitic nematodes. When recovered from compost or isopods, which worms may use as vectors to shuttle between food sources, *C. elegans* is frequently found in the dauer stage (Félix and Duveau, 2012). In contrast, proliferating populations of nematodes can be found in microbe-dense environments, particularly rotting fruits or vegetation. A study by Samuel et al. characterized the natural bacterial microenvironment of *C. elegans* (Samuel et al., 2016). The most prevalent bacterial phyla in decaying apples were Proteobacteria, Bacteroidetes, Firmicutes, and Actinobacteria. The Enterobacteriaceae family within Proteobacteria encompassed the most abundant genera detected, while *Lactococcus, Lactobacillus, Acetobacter, Gluconobacter*, and *Gluconoacetobacter* were also quite prevalent. In some samples, *Escherichia* spp. were also identified, though they were not particularly abundant. Considering the immensity of bacterial diversity coming from 29 extant phyla (Yarza et al., 2014), there is considerable overlap in the microbial environments of parasitic and free-living nematodes. Experiments assessing the effect of *C. elegans* on an experimental microbiota could be designed, whereby the nematodes are introduced into a defined microbial environment and followed over time to assess changes to bacterial constituents. Such studies would be analogous, albeit profoundly simplified, to studies assessing gut microbial changes in helminth infections and could shed light on the underlying mechanisms contributing to compositional alterations. Akin to some reports of parasitic nematodes, observations from rotting fruit suggest that growth of *C. elegans* is enhanced amongst a simpler microbiota (Samuel et al., 2016).

Many studies have demonstrated the susceptibility of the *C. elegans* intestine to a multitude of pathogens (Couillault and Ewbank, 2002), including *Salmonella enterica* serovar Typhimurium (*S. typhimurium*) (Lee et al., 2015), *Enterococcus faecalis* (Sim and Hibberd, 2016), *Burkholderia* spp. (Lee et al., 2013), *Staphylococcus aureus* (JebaMercy and Balamurugan, 2012), *Proteus mirabilis* (JebaMercy and Balamurugan, 2012), and *Pseudomonas aeruginosa* (Dai et al., 2015). Beyond posing a threat to the nematode intestine, bacteria may also damage and infect the nematode surface. In experimental settings, *Yersinia* spp. can form biofilms that block the oral cavity of the worms, resulting in starvation and death (Tan and Darby, 2004). Wild nematodes are subject to cuticular infection by *Microbacterium nematophilum* (O'Rourke et al., 2006) and the fungal pathogen *Drechmeria coniospora* (Engelmann et al., 2011). Additionally, *Elizabethkingia* are capable of digesting the nematode cuticle components keratin and collagen (Riffel et al., 2003; Félix and Duveau, 2012). *C. elegans* is also infected by intracellular pathogens such as the microsporidian parasite *Nematocida parisii* (Troemel et al., 2008) and the Orsay virus (Félix et al., 2011). Though studies have demonstrated the ability of bacteria to colonize parasitic nematodes, no information is available concerning which microbes might be beneficial or harmful for them. In contrast, numerous studies in *C. elegans* have shown beneficial and harmful effects of specific bacterial strains. Alphaproteobacteria and *Lactococcus* spp. tend to be beneficial and promote *C. elegans* proliferation while Bacteroidetes and Gammaproteobacteria seem to impair nematode physiology and induce stress/immune responses (Samuel et al., 2016). It stands to reason that similar interactions exist between bacteria and parasitic nematodes and that the composition of the host microbiota would then influence the health of parasitic nematodes in the gut. Interestingly, nematode fecundity is subject to various stresses; the host immune response can decrease helminth fecundity, exemplified by reduced egg production by *H. p. bakeri* during a type 2 helper T cell-mediated immune response (Strandmark et al., 2016). As bacteria influence *C. elegans* fecundity (Szewczyk et al., 2006), it is possible that communities within the microbiota may also influence the reproductive capacity of parasites. Larger populations of proliferating *C. elegans* are found in more highly degraded apples containing larger microbial populations, thereby providing more nutritional sources for the nematodes. Also, apples with higher populations of worms had microbiota which were more similar to each other than apples with lower/no worms. Apples containing proliferating populations of *C. elegans* also had significantly fewer bacterial species and diversity than apples with non-proliferating populations. Most importantly, proliferation was associated with Alphaproteobacteria and the absence of potential pathogens from Gammaproteobacteria and Bacteroidetes. Perhaps there is a general preference by nematodes for decreased abundance of several Bacteroidetes organisms, given the observations in parasite-host microbiota studies discussed previously. As Lactobacillaceae are more predictive of successful *H. p. bakeri* establishment in mice (Reynolds et al., 2014), Enterobacteriaceae and Acetobacteraceae are predictive for larger proliferating *C. elegans* populations in rotting apples, while Gammaproteobacteria show an inverse relationship (Samuel et al., 2016). It is not fully understood how intestinal nematodes establish in specific habitats such as the cecum and colon in the case of Trichurids, or the small intestine in the case of Ascarids and *H. p. bakeri*; however, some speculate the choice is based on the abundance of nutrients or the relative lack of harmful stimuli (Davey, 1964; Bansemir and Sukhdeo, 1994, 2001). While determining the nature of habitat selection in these settings may prove experimentally elusive, the available data suggest that parasitic nematodes, as with their free-living counterparts such as *C. elegans*, have advantageous associations with some microbial species and detrimental interactions with others.

### Colonization of nematodes by microbes

All animals, including nematodes, live in association with a multitude of other species. The host-microbe relationship has gained a lot of attention in recent studies, with various reciprocal interactions ranging from parasitism, to commensalism and mutualism, seen as potent forces guiding the co-evolution of species. In addition to influencing the rich and diverse microbial environments that they inhabit, nematodes inevitably also harbor their own microbiota that is likely essential for normal development and physiology, as is the case for other organisms. Though the data are limited, studies have documented the ability of bacteria to colonize *Ascaris, H. p. bakeri*, as well as *C. elegans*.

Earlier studies demonstrated nematode-associated bacterial communities in *A. suum* (Hsu et al., 1986; Shahkolahi and Donahue, 1993). One study aimed at identifying the source of serotonin present in nematodes, which generally lack the enzymes necessary for *de novo* synthesis of serotonin from tryptophan, revealed 4 × 10^9^ bacteria per gram of nematode intestine (Hsu et al., 1986). In addition to *E. coli, Enterobacter, Klebsiella, Acinetobacter, Citrobacter, Pseudomonas, Aeromonas*, and *Shigella* were identified among gram-negatives while *Staphylococcus, Streptococcus, Corynebacterium*, and *Bacillus* were identified amongst gram-positive organisms. The species identified suggest that the nematode's intestinal microbiota may have been derived from that of the host. Another study demonstrated that antibiotic treatment of *ex vivo* cultured *A. suum* reduced, but did not eliminate, the bacterial load carried by the worms and determined that the posterior portion of the intestine harbored the highest number of culturable bacteria (Shahkolahi and Donahue, 1993). Studies that employ more modern methods while sampling site-specific host- and parasite-associated microbial communities could greatly enhance our understanding of the selectivity and development of an *Ascaris*-specific microbiota.

Another study showed that *A. lumbricoides* nematodes isolated from cholera patients were colonized by *Vibrio cholerae* (Nalin and McLaughlin, 1976). Worms were retrieved from patients after being spontaneously passed or after de-worming and a majority of worms were colonized by *V. cholerae* of the same serotypes found in stool samples of cholera patients. Serial sectioning of the worms revealed colonization along the entire length of the intestine, from oral cavity to anus. The bacteria were recovered and viable even after worms were in culture in saline for 6 days. The authors reported retrieval of one hookworm which also revealed two colonies of bacteria which were not *V. cholerae*. The authors reasoned that pathogenic bacteria may survive inside parasites when parasites are passed in the stool, as happens commonly during a cholera infection, thereby contributing to environmental spread of microbial pathogens. Further, the authors discussed the possibility of pathogenic microbes reaching the reproductive tract of female nematodes during copulation and adhering to egg shells which may also serve as a vehicle for transmission. There was no indication in this study that *V. cholerae* are pathogenic to Ascarids and further study in this area could reveal interesting associations between different enteric infections. Taken together, these findings highlight the clinical relevance of understanding interactions between intestinal nematodes and environmental microbes.

Walk et al. provided evidence for a *H. p. bakeri*-associated microbiota when they sampled L3 and adult nematodes (Walk et al., 2010), finding only 28 16S sequences obtained from larvae, concluding that they are associated with few bacteria; however, the L3-associated microbiota was completely unique and consisted of 6 bacterial families, unlike the adult-associated microbiota which was very similar to the ileum of the murine host. As *H. p. bakeri* hatch outside of the host, they likely enter their hosts with a distinct microbiota which changes over time in the new environment. This L3-specific microbiota may also serve as a source of non-indigenous microbes for the mammalian host.

A special microbe-helminth relationship is also well documented for filarial nematodes in which the gram-negative intracellular bacterium *Wolbachia* is obligatory for normal larval growth and development, embryogenesis, and survival of adult worms (Taylor et al., 2005). Such endosymbiotic relationships probably emerged from ancestral infections of the host nematode by free-living bacteria, concomitant with gene losses and genome rearrangements on both sides during coevolution (Masson et al., 2016). So far, such an endosymbiotic relationship has not been detected in other parasitic nematode species, but parallels to other microbe-nematode partnerships may be drawn. For example, *Ascaris* requires and absorbs Vitamin B12 from microbial sources (Zam and Martin, 1969). *Wolbachia* displays cell tropism and is restricted to somatic tissues in adult male worms, whereas females also harbor bacteria in the germline (Kozek, 1977; Taylor et al., 1999). The level of infection varies substantially during filarial development, where shortly after transmission to the mammalian host a dramatic increase in the bacterial population occurs (McGarry et al., 2004). As bacterial loads within individual worms differ, Taylor et al. hypothesized that higher levels of *Wolbachia* infection within a worm may potentially confer selective advantages in terms of filarial development or fecundity (Taylor et al., 2013). Transcriptomic analysis of the *Wolbachia* genome of *Onchocerca ochengi* indicated that *Wolbachia* may have a mitochondrion-like function in the soma, generating ATP for the nematode host (Darby et al., 2012). Hence, recent trials aim at utilizing antibiotics such as tetracycline (Hoerauf et al., 1999), and other chemotherapeutics targeting *Wolbachia* as a novel tool for the treatment of filarial infection and disease, reviewed in Taylor et al. (2014).

*C. elegans* nematodes isolated from the wild are observed to harbor live bacteria in their intestine which may exist as commensals or which may proliferate and cause obstruction and pathology of the worm gut (Félix and Duveau, 2012). Worms isolated from the wild also carry a distinct and diverse microbiota when compared to other nematode species in the same environment (Dirksen et al., 2016). Dirksen et al. reported a microbiota rich in unclassified Proteobacteria from the family Enterobacteriaceae, as well as members of the genera *Pseudomonas, Stenotrophomonas, Ochrobactrum*, and *Sphingomonas*. When isolated worms were enriched on plates of *E. coli* OP50, their microbiota maintained considerable similarity to freshly isolated samples despite 3 weeks in culture on *E. coli* plates, suggestive of a *C. elegans*-specific microbiota and closely developed microbial-host community. This study did not mention any changes in overall microbial population sizes over time. In the same study, a subset of 14 bacterial isolates were chosen in order to cultivate nematodes on an experimental microbiota with bacterial frequencies mirroring the nematode-associated microbiota of worms isolated from the wild. Three genotypes of *C. elegans*, including two natural isolates (MY316 and MY379) and the laboratory N2 strain, were cultured from hatched sterilized eggs through to adulthood. The investigators found significant influences of genotype and life stage on the microbial composition of the nematodes. Interestingly, certain bacteria including *Ochrobactrum* MYb71 and *Stenotrophomonas* MYb57 were enriched in the nematode samples relative to the agar plates. The effect was quite pronounced in the case of *Ochrobactrum* which was present in only trace amounts in the agar plates but represented as much as 20% of the nematode-associated bacterial community. *Ochrobactrum* was also able to persist in the intestine even under starvation conditions without being used as a food source or eliminated during the ensuing stress response, which can include upregulation of antimicrobial effectors such as lysozymes (Uno et al., 2013). This bacterial strain may be a prominent symbiont for *C. elegans* as it seems to use the nematode as an environmental niche in the absence of apparent fitness costs to the worm. The experimental microbiota was also shown to enhance growth and nematode population size relative to worms cultured on *E. coli*. While detailed analysis of this sort is absent for parasitic nematodes, the ability of microbes to colonize these worms supports the idea of worm-associated microbial communities, providing benefits similar to those seen in *C. elegans* and other organisms, such as supplying nutrients and providing protection from pathogens (Cabreiro and Gems, 2013; Watson et al., 2014; Lee et al., 2015; Dirksen et al., 2016).

## The influence of bacteria on parasitic nematodes

Living in intimate association with microbes, nematodes are subject to diverse microbial influences. Beneficial effects of microbes on nematodes may include nutrition, promotion of longevity, protection from infection by other microbes, and contributions to a hospitable environment (Cabreiro and Gems, 2013; MacNeil and Walhout, 2013). From the perspective of mammalian hosts, the question arises whether a specific microbial environment might significantly influence susceptibility to helminth infection. Further, microbes might also be a source of competition, stress, and disease for nematodes.

The microbial environment can profoundly impact establishment and propagation of parasitic nematode infections by influencing egg hatching and reproductive success. *T. muris* eggs may require the presence of selected bacterial species to induce hatching, as demonstrated using murine cecal explants as well as *E. coli, S. typhimurium, S. aureus*, and *P. aeruginosa* (Hayes et al., 2010). Live bacteria appear to be necessary for these effects as heat inactivation prevented hatching, while bacteriostatic gentamycin treatment did not. It was proposed that physical contact between the bacteria and eggs is required as Type 1 fimbriae facilitate *E. coli*-induced hatching, though additional mechanisms likely exist. The authors also reported that mice pre-treated with antibiotics had significantly lower worm burdens at day 21 pi compared to untreated animals, indicating a significant role for bacterial communities in parasitic nematode establishment. Interestingly, bacteria-induced *T. muris* egg hatching seems to occur efficiently with members of Proteobacteria (*E. coli*) and Firmicutes (*Enterococcus caccae, Streptococcus hyointestinalis, Lactobacillus reuteri*, and *Lactobacillus amylovorus*). It is possible that many different bacterial species contribute via different mechanisms for optimal *T. muris* egg hatching (Vejzagić et al., 2015).

Lactobacillaceae abundance in the duodenum positively correlates with susceptibility to *H. p. bakeri* infection (Reynolds et al., 2014). Low-level vancomycin treatment prior to nematode infection did not significantly reduce total bacteria but elevated abundance of Lactobacillaceae and Enterobacteriaceae while reducing *Eubacterium*/*Clostridium* species in the fecal microbiota. This was associated with *H. p. bakeri* persistence in the host. *H. p. bakeri* infection also elevated duodenal Lactobacillaceae and Enterobacteriaceae. Administration of *Lactobacillus taiwanensis* enhanced susceptibility to *H. p. bakeri* infection and worm fecundity, thought to be due to the induction of immunosuppressive regulatory T cells. This study demonstrated a reciprocal interaction whereby *Lactobacillus* spp. promote nematode establishment which then promote growth of *Lactobacilli*. Similar to *T. muris* infections discussed above, studies in germfree mice revealed higher *H. p. bakeri* worm burdens in conventionally raised mice, implicating the host microbiota as a key part of the parasite's environmental niche (Wescott, 1968). Bacterial populations also influence the host's immune status which can have a profound influence on the intestinal environment for parasite establishment, though specific immune variables and bacterial species are still being identified (Cattadori et al., 2016). These data suggest not only that the microbiota is essential for parasite development, but also that particular bacteria facilitate helminth establishment.

While *C. elegans* and other free-living nematodes are known to subsist on microbes, the food sources of intestinal nematodes are less well established. The digested remains of yeasts can be found in the intestines of *C. elegans* isolated from the wild, demonstrating the capability of these nematodes to use eukaryotic cells as a food source in addition to bacteria (Félix and Duveau, 2012). There is corresponding evidence suggestive of consumption of intestinal epithelia by *A. suum* in pigs as well as *H. p. bakeri* in mice (Davey, 1964; Bansemir and Sukhdeo, 1994). Freshly isolated *Ascaris* nematodes were found to accumulate eukaryotic cellular material in their buccal cavities, thought to be of host origin (Davey, 1964). Labeling studies sought to determine whether *H. p. bakeri* accumulates ingesta, host blood, or host tissue material and found an accumulation of host tissue components rather than blood or ingesta (Bansemir and Sukhdeo, 1994). These studies did not assess the intestinal contents of the worms and these data do not preclude the ability of intestinal nematodes to digest bacterial cells, especially considering bacteria are counted amongst the intestinal contents of *A. suum* and *A. lumbricoides* as discussed previously. Further insights using modern methods could be provided by studies designed to specifically assess uptake and digestion of microbes by intestinal nematodes.

Bacteria can promote or hinder nematode proliferation by various means. From serving as direct food sources or by producing essential nutrients, microbes are required for nematode growth. Furthermore, specific species are well documented to promote helminth establishment while themselves being reinforced by nematodes, as discussed. One could imagine that intentional manipulation of microbial variables could influence parasite establishment and might possibly impact how parasitic diseases are treated in the future. In the case of *C. elegans*, characterizing its interactions with microbes beyond *E. coli* OP50 serves to improve this powerful model and enhances its utility to investigators with diverse objectives. Together, these diverse systems complement each other in elucidating the varied interactions between nematodes and microbes, thereby setting a stable foundation upon which future research can build.

## Sensing the microbial environment

Nematodes are confronted with a tremendous and highly diverse microbial environment, presenting many infectious challenges. Alterations in the intestinal microbiota during helminth infection, coupled with studies demonstrating distinctly beneficial or harmful outcomes to *C. elegans* physiology in response to various bacteria, illustrate the ability of nematodes to sense their microbial environments and possibly discriminate between microbial species. Parasitic nematodes must have evolved particular strategies to overcome colonization by microbes, likely employing selected antibacterial defense mechanisms to coexist with the host microbiota. *C. elegans* has become an important model organism for the study of innate immune defense against pathogenic bacteria (Schulenburg et al., 2004; Irazoqui et al., 2010). The *C. elegans* immune system is seemingly of ancient origin, and there exist homologs of nearly all of its components in other organisms, including humans (Schulenburg et al., 2008). Consequently, sensing of the microbial environment might follow conserved or comparable mechanisms in parasitic nematodes as well.

*C. elegans* uses different protective mechanisms when confronted with potential pathogens, such as avoidance behavior (Meisel and Kim, 2014) and activation of stress and immune responses (Samuel et al., 2016) leading to the production and release of defense molecules with antimicrobial activities (Mallo et al., 2002). Identification of microbe-associated molecular patterns is followed by signal transduction via several signaling cascades, culminating in transcriptional alterations, reviewed in Rosso et al. (2013) and Kim and Ewbank (2015). While the pathogen-recognition receptors responsible for initiating immune responses are not entirely known, candidates include F-box proteins, lectins, follicle stimulating hormone receptor homolog-1, scavenger receptors, and the Toll-like receptor TOL-1 (Kim and Ewbank, 2015). Stress and immune responses illustrate an overlap between microbial and abiotic stresses; the main responses involved include: autophagy, insulin-like receptor (DAF-2), mitogen-activated protein kinases (MAPK), transforming growth factor-β-like (DBL-1), programmed cell death pathways, and unfolded protein responses (UPR) (Kim and Ewbank, 2015). Perhaps surprisingly, *C. elegans* lacks a homolog of NF-κB, an essential transcription factor in the innate immune response of many invertebrate and vertebrate species (Vallabhapurapu and Karin, 2009). Other proteins regulating transcription of infection and stress-modulated genes have been identified, including cyclic AMP-dependent transcription factor-7 (ATF-7), forkhead box O (FOXO) ortholog DAF-16, GATA transcription factors (ELT-2, ELT-3), helix loop helix-30 (HLH-30), heat-shock factor-1 (HSF-1), NF-E2-related factor SKN-1, signal transducer and activator of transcription STA-2, X-box binding protein-1 (XBP-1), and basic leucine zipper domain transcription factor ZIP-2 (Kim and Ewbank, 2015). As indicated, the intricacies of these responses have been reviewed elsewhere; as such, only examples of particular interest are highlighted here.

### Pathogen recognition

Infectious risks could be mitigated and resources preserved if pathogens were simply not encountered. Avoidance behavior is facilitated by the sole *C. elegans* toll-like receptor, TOL-1 (Pujol et al., 2001), and is characterized by the detection of specific microbial products, such as serrawettin W2 produced by *Serratia marcescens*, resulting in worms migrating away from the offending agents (Pradel et al., 2007). Unlike other organisms, *C. elegans* may not rely heavily on toll-like signaling for immune defense, though reports on this are conflicting (Pujol et al., 2001; Couillault et al., 2004; Tenor and Aballay, 2008). Other mechanisms such as aerotaxis also contribute to pathogen avoidance, as demonstrated by *C. elegans* avoidance of *P. aeruginosa* (Reddy et al., 2009). Lectins, carbohydrate-binding proteins implicated in multiple facets of immunity in a diverse range of species, may sense or neutralize microbes (Zelensky and Gready, 2005). Finally, there is evidence for an important role for scavenger receptors such as cell death abnormal-1 (CED-1) and scavenger (SCAV) proteins in pathogen recognition and resistance (Nicholas and Hodgkin, 2004; Means et al., 2009). Comparable studies of differential activation of recognition receptors in parasites by microbial communities from different intestinal regions might provide insights into how parasitic nematodes recognize threats and whether or not activation of such pathways impacts their niche selection.

### Surveillance immunity

In addition to sensing microbes and their toxins, organisms induce immunity-related genes due to disruptions of core physiologic processes by pathogens. *C. elegans* employs this surveillance immunity in response to inhibited translation, cellular damage, and mitochondrial stress, all of which can result from infection and exposure to microbial toxins (Pukkila-Worley, 2016). Transcriptional responses to these stresses include activation of immune signaling and xenobiotic metabolic pathways (Pukkila-Worley, 2016).

Toxins produced by several microbes, including diphtheria toxin from *Corynebacterium diphtheria* (Collier, 2001), cholix toxin from *Vibrio cholerae* (Jørgensen et al., 2008), and exotoxin A (ToxA) from *P. aeruginosa* (Dunbar et al., 2012), interfere with protein translation. When non-pathogenic *E. coli* engineered to express ToxA are fed to *C. elegans*, immune pathways required for resistance to the toxin, especially MAPK pathways, are upregulated by the worms (McEwan et al., 2012). Also, the aminoglycoside antibiotic hygromycin B blocks elongation of the amino acid chain during protein translation like ToxA (McEwan et al., 2012). Taken together, *C. elegans* likely responds to translation inhibition by ToxA rather than to the protein itself. An RNAi screen showed that disrupting core processes, including translation, activatesbasic leucine zipper domain transcription factor ZIP-2-dependent immune signaling, similar to responses seen during *P. aeruginosa* infection of *C. elegans* and following exposure to ToxA (Dunbar et al., 2012). More recently, another transcription factor called CCAAT-enhancer-binding-protein-2 (CEBP-2) was identified to act in concert with ZIP-2 to promote defense by *C. elegans* against *P. aeruginosa*, ToxA, and in response to inhibition of translation and other core processes (Reddy et al., 2016).

In addition to surveillance of disrupted physiologic processes, *C. elegans* also induces immune responses after injury of its epidermis by microbial infection and sterile wounding (Pujol et al., 2008a). During infection with *D. coniospora*, a fungal pathogen which damages the nematode cuticle, 4-hydoxyphenyllactic acid (HPLA) acts as a damage-associated molecular pattern recognized by the G protein-coupled receptor DCAR-1 which is required for AMP expression during fungal infection (Zugasti et al., 2014). Another study showed that epidermal damage liberates the STAT-like transcription factor STA-2, triggering AMP production (Zhang et al., 2015). These findings exemplify the ability of epithelial barriers to surveil physical damage and respond by activating innate immune pathways.

Microbial infection can often lead to mitochondrial stress; a survey of *C. elegans* responses to bacteria isolated from the nematode's natural environment found that 101 strains of the >550 isolates tested induced the mitochondrial stress reporter promoter *hsp-6::GFP* (Samuel et al., 2016). Interestingly, mitochondrial disruption also induces drug-detoxification enzymes belonging to the cytochrome P450 superfamily (CYPs) and enzymes involved in glucuronidation (Liu et al., 2014) as well as infection response gene-1 (*irg-1*) which is also induced during *P. aerugionsa* infection in a ZIP-2-dependent manner (Estes et al., 2010; Dunbar et al., 2012). Another toxin of *P. aeruginosa*, pyoverdin, disrupts the mitochondria of *C. elegans*. The nematodes respond by a specialized form of autophagy called mitophagy, whereby damaged mitochondria are degraded to resist *P. aeruginosa*-mediated killing (Kirienko et al., 2015).

By sensing various forms of cellular stress and damage, *C. elegans* is able to launch an integrated response which promotes survival and longevity (Pukkila-Worley, 2016). These responses can be induced by microbes and xenobiotics, activating immune and detoxification pathways (Melo and Ruvkun, 2012; Pukkila-Worley et al., 2014; Pukkila-Worley, 2016). As helminths may employ similar strategies to deal with microbial and xenobiotic stresses, the overlap between immune and drug detoxification responses is of particular interest. Various contributing factors have been identified for the development of anthelmintic resistance, including resistance alleles in detoxification genes, parasites not exposed to treatments, and underdosing (Vercruysse et al., 2011). The concept of surveillance immunity raises the notion that elements within the host microbiota prime helminths to resist anthelmintic treatments. Whether certain microbial species of the host intestine can promote drug resistance by activating xenobiotic detoxification within intestinal nematodes has not yet been studied.

### Signal transduction pathways and transcriptional responses

As illustrated by many studies, MAPK pathways have a fundamental function in *C. elegans* stress and immune responses, including the p38 PMK-1 and extracellular signal-regulated kinase (ERK) pathways. Mutations at multiple levels of the NSY-1-SEK-1-PMK-1 pathway increase susceptibility to *P. aeruginosa* without impacting growth on *E. coli* OP50 (Kim et al., 2002). PMK-1 is activated by the toll-interleukin-1 receptor domain adaptor protein TIR-1 which is required for resisting killing by numerous microbial pathogens (Couillault et al., 2004). Phosphorylation of the ATF-7 transcription factor by PMK-1 activates transcription of lectins and candidate antimicrobial peptides (AMPs), conferring intestinal resistance to various species, including *P. aeruginosa, S. marcescens*, and *E. faecalis* (Shivers et al., 2010). Small molecule-mediated stimulation of the PMK-1 pathway and subsequent activation of ATF-7 can also increase lifespan in the presence of *E. faecalis* (Pukkila-Worley et al., 2012). Growth of *C. elegans* on soil-derived non-pathogenic bacteria, *Bacillus megaterium* and *Pseudomonas mendocina* also enhanced resistance to *P. aeruginosa* in a PMK-1 dependent fashion (Montalvo-Katz et al., 2013). Additionally, the PMK-1 pathway is active in epidermal infections as shown by induced expression of AMPs in response to *D. coniospora* as well in response to epidermal injury (Pujol et al., 2008a,b). Another MAPK pathway involving ERK signaling has been shown to be protective against rectal infection by *M. nematophilum*, (Gravato-Nobre et al., 2005). Based on these observations, one could speculate that MAPK pathways are also essential for helminth survival amongst numerous microbes.

Insulin-like signaling via DAF-2 highlights the intersection between immunity and metabolism. DAF-2 signaling inhibits DAF-16 activation by preventing its localization to the nucleus. Loss of function mutations of *daf-2* confer pathogen resistance and longevity by allowing activation of the DAF-16 transcription factor (Kenyon et al., 1993; Garsin et al., 2003). DAF-16 activation also increases resistance to non-microbial insults, such as heat stress and oxidative stress (Barsyte et al., 2001). Intriguingly, *C. elegans* can be primed to withstand pathogens and heat stress when exposed to pathogens during development, increasing the worm's lifespan (Leroy et al., 2012). DAF-16 activation appears to increase antimicrobial gene transcription as well as stress and detoxification genes, placing this transcription factor at the nexus of stress and immune signaling (McElwee et al., 2003). Autophagy, a lysosomal degradation pathway, is also implicated in longevity and its inhibition increases intracellular replication of *S. typhimurium* in *C. elegans* intestinal epithelia (Jia et al., 2009).

As intestinal nematodes are typically much larger than *C. elegans*, with female *Ascaris* worms growing up to 35 cm in length (Dold and Holland, 2011), the surface area available for attachment and infection by microbes is extensive. Furthermore, these nematodes are much longer-lived than *C. elegans*. Stress responses, especially autophagy, may be of particular importance in these organisms, though this area remains virtually unexplored. An intriguing study by Voronin *et al*. identified autophagy as a bactericidal mode of action in filarial worms targeting the bacterium *Wolbachia* (Voronin et al., 2012). The activation of autophagy coincided with the onset of rapid bacterial growth and expansion, showing that in spite of their mutualistic association, the nematode may recognize *Wolbachia* as a stressor and respond to regulate bacterial abundance (Taylor et al., 2013). Further studies assessing antibacterial responses by intestinal nematodes are largely lacking; however, two reports have shed some light on the issue. Adult female *A. suum* nematodes challenged with heat-inactivated *E. coli* respond by increasing transcription of two AMP families, the *Ascaris suum* antibacterial factors (ASABF) and the Cecropins (Pillai et al., 2003, 2005). Worms were challenged by injection of heat-killed bacteria into the pseudocoelom and appeared to demonstrate tissue-specific responses. While these data suggest an inducible defense system in *A. suum*, it would be relevant to study tissue-specific AMP expression using live bacterial cultures introduced externally, without wounding the worms, coupled with modern transcriptomic methods. Such an experiment would preserve pathogen-associated molecular patterns without invoking pathogen-independent, epithelial-damage responses, thereby allowing identification of transcriptional changes induced specifically by infection.

## Effectors of innate immune signaling in nematodes

After sensing microbes, typical responses by nematodes include the production and release of factors possessing antimicrobial activity (Table 3). Among the different effectors of innate immunity, AMPs constitute the most ancient gene-encoded antimicrobial tools of eukaryotes (Zasloff, 2002). Nematodes produce different families of AMPs, many of which have been well characterized in *C. elegans*, while some have also been identified in parasitic nematodes. Antimicrobial activity has been experimentally demonstrated for many of these factors. AMPs can be classified based on their amino acid constituents and structural characteristics: α-helices, β–sheets, extended, and looped (Melo et al., 2009). Many of the best-characterized AMPs tend to be small, cationic, amphipathic molecules thought to act by membrane disruption. While many candidate immune effectors have been proposed in the literature for *C. elegans*, reviewed in Kim and Ewbank (2015), here we focus on effectors with corresponding data from parasitic nematodes. AMPs such as antibacterial factors and cecropins are discussed along with larger proteins involved in the immune response including lectins, nemapores, and lysozymes. We will then conclude this section by discussing antimicrobial activities of helminth-derived products.

**Table 3:** **Selected antimicrobial molecules of nematodes**.

	**Parasites**			**Free-living**	
	***Ascaris* spp**.	***Trichuris* spp**.	***H. p. bakeri***	***C. elegans***	**References**
Antibacterial factors	Yes	–	–	Yes	Kato et al., 2002; Andersson et al., 2003; Pillai et al., 2003
Cecropins	Yes	–	–	–	Andersson et al., 2003; Pillai et al., 2005
Lectins	Yes^*^	–	Yes	Yes^*^	Cuperlović et al., 1987; Mallo et al., 2002; Harcus et al., 2009; Engelmann et al., 2011; Wang et al., 2013; Miltsch et al., 2014
Lysozymes	Yes	–	Yes	Yes	Mallo et al., 2002; O'Rourke et al., 2006; Schulenburg and Boehnisch, 2008; Hewitson et al., 2011; Tarr, 2012; Wang et al., 2013; Gravato-Nobre et al., 2016
Nemapores	Yes	Yes	–	Yes	Bányai and Patthy, 1998; Coolon et al., 2009; Roeder et al., 2010; Tarr, 2012
Nematode products	Tissue extracts, pseudocoelomic fluid	ESP	–	–	Wardlaw et al., 1994; Kato, 1995; Abner et al., 2001

Yes, detected; -, not detected;

* *, lectin-like activity detected; gray shading, demonstrated bactericidal activity*.

### Antibacterial factors

First identified in *A. suum*, ABFs are present in at least 25 nematode species, including seven ASABFs produced by *Ascaris* and six ABFs produced by *C. elegans* (Tarr, 2012). Sequence analysis reported by Tarr shows that other parasitic nematode species expressing ABFs include the human hookworm *N. americanus*, the ruminant nematode *Haemonchus contortus*, and the rodent model parasite *N. brasiliensis* (Tarr, 2012). Body fluid isolated from the pseudocoelem of *A. suum* demonstrated bactericidal activity, particularly against gram positive organisms (Wardlaw et al., 1994; Kato, 1995). Kato *et al*. isolated ASABF-α (Kato and Komatsu, 1996) and subsequently demonstrated broad-spectrum antibacterial and weak antifungal activity of a recombinant form of the peptide (Zhang et al., 2000). The observed activity was rapid, killing *S. aureus* in under 1 min, and could be inhibited by increasing salt concentrations (Zhang et al., 2000). Andersson *et al*. isolated ASABF-β and γ from *A. lumbricoides* which also showed antimicrobial activity, especially against gram positives (Andersson et al., 2003). ABFs were the first AMPs described in *C. elegans*, based on sequence similarity to their Ascarid counterparts and also demonstrated antimicrobial activity (Kato et al., 2002). ABFs belong to the cysteine-stabilized-αβ (CS-αβ) group of AMPs, are cationic, and contain a cysteine-stabilized α-helix and two β-sheets (Kato and Komatsu, 1996; Tarr, 2012). Transcripts for ASABFs have been detected in all tissues of *A. suum*, with some members upregulated in the body wall, intestine, and ovaries after bacterial challenge (Pillai et al., 2003). Expression patterns and anatomical localization of ABFs in *C. elegans* are indicative of roles in defense and digestion (Kato et al., 2002). For example, ABF-1 and 2 are detected in the pharynx, and ABF-1 and 3 in the intestine of *C. elegans*. The expression of some ABFs is inducible by different pathogens: ABF-2 by *S. typhimurium* (Alegado and Tan, 2008), ABF-1 and 2 by *Cryptococcus neoformans*, and ABF-3 by *S. aureus* (Alper et al., 2007).

### Cecropins

In contrast to ABFs which seem to be widely distributed amongst nematodes, the cecropin family of AMPs has only been found in three Ascarids: *A*. *suum, A. lumbricoides*, and *Toxocara canis* (Pillai et al., 2005; Tarr, 2012). The first described nematode cecropin, Cecropin P1, was originally thought to be of porcine origin due to its isolation from pig intestine (Lee et al., 1989). Subsequently, its production was correctly attributed to *A. suum* (Andersson et al., 2003). Andersson et al. also isolated Cecropin P1 from *A. lumbricoides* and demonstrated antibacterial activity against gram negative organisms (Andersson et al., 2003). Pillai et al. demonstrated broad-spectrum antimicrobial activity of all four *Ascaris* cecropins as well as bacterial inducibility as detected for ASABFs (Pillai et al., 2005). Cecropins are typically 31–39 amino acids in length, strongly basic, cationic, α-helical peptides which are thought to disrupt microbial membranes by first laying on the membrane surface before reorientation and insertion into the membrane leading to disrupted lipid packing and subsequent membrane disintegration (Sipos et al., 1992; Gazit et al., 1995).

### Lectins

Proteins containing lectin domains are conserved across Metazoans, including nematodes (Zelensky and Gready, 2005). Harcus et al. isolated one C-type lectin (CTL) from *H. p. bakeri* and two from *N. brasiliensis* (Harcus et al., 2009). Moreover, lectin-like carbohydrate-binding activity has previously been reported in the intestine of *A. suum* (Cuperlović et al., 1987) and lectins have been identified in excreted and secreted products (ESPs) of *A. suum* larvae (Wang et al., 2013). In general, CTLs are capable of binding carbohydrates and function in pathogen recognition and neutralization (Zelensky and Gready, 2005). CTLs from *H. p. bakeri* and *N. brasiliensis* are predominantly expressed by gut-dwelling adults rather than larvae; while Harcus et al. made a compelling case for host-immunomodulation by their reported CTLs, a dual role in pathogen neutralization or recognition is worth testing for parasite-derived lectins (Harcus et al., 2009). Parasite-derived CTL molecules localize in the nematode cuticle but could also be found in ESPs (Page et al., 1992). The CTLs detected in parasitic nematodes were most similar to the *C. elegans*-CTLs *clec-48*, -*49*, and -50. *Clec-50* is expressed in the *C. elegans* intestine and upregulated in response to the bacterium *S. marcescens* (Mallo et al., 2002). Though Harcus et al. did not report antimicrobial activity for the parasite-derived molecules, studies in *C. elegans* suggest they may have a role in defense. In addition to *clec-50, clec-49* is also upregulated in response to *S. marcescens* (Engelmann et al., 2011) and mutants deficient in this protein and another CTL, *clec-39*, have increased susceptibility to, and reduced survival and fecundity during, *S. marcescens* infection compared to wild-type worms (Miltsch et al., 2014). Furthermore, recombinant *clec-39* and *clec-49* bind *S. marcescens* in the absence of bactericidal activity (Miltsch et al., 2014). Additionally, in response to *M. nematophilum*, CTLs were found to be the most up-regulated protein class in *C. elegans* (O'Rourke et al., 2006). While nematode-derived lectins have not yet been shown to possess bactericidal activity, the mammalian lectin RegIIIγ is antibacterial and promotes commensalism by maintaining segregation between the microbiota and the host epithelium in mice (Vaishnava et al., 2011). Though many questions remain, the limited evidence available is suggestive of a role for lectins during infectious challenges and it is possible that parasite-derived lectins may confer resistance to different bacteria as has been demonstrated for *C. elegans*.

### Lysozymes

Lysozymes are polysaccharide hydrolases, targeting bacterial peptidoglycan leading to cell lysis (Ellison and Giehl, 1991; Monzingo et al., 1996), and have been widely found in different nematode species. Lysozymes are encoded in the genomes of Ascarids, *N. americanus, H. contortus, N. brasiliensis, Brugia malayi*, and *Wuchereria bancrofti*, though missing from the Trichurids included in the sequence analysis by Tarr (2012). Lysozymes were also detected in the ESPs of *A. suum* (Wang et al., 2013) and *H. p. bakeri* (Hewitson et al., 2011), though further study is required to determine antibacterial activities of these helminth-lysozymes. 15 lysozymes were detected in *C. elegans*, divided into 10 protist-type (*lys-1–lys-10*) and five invertebrate-type (*ilys-1–ilys-5*), so named for sequence similarities to other organisms (Schulenburg and Boehnisch, 2008). Some lysozymes are markedly upregulated by *C. elegans* in response to bacterial infection (Mallo et al., 2002; O'Rourke et al., 2006; Gravato-Nobre et al., 2016). Lysozymes expressed in the *C. elegans* intestine (Schulenburg and Boehnisch, 2008) have been proposed to act in concert with caenopores (discussed in the next section) in digestion and immunity (Bányai and Patthy, 1998). For example, the *C. elegans* invertebrate lysozyme ILYS-3 is needed for physiological pharyngeal grinder function and for defense against bacterial pathogens (Gravato-Nobre et al., 2016). It was also shown that ILYS-3 is induced by danger signals generated both by bacterial pathogens and starvation (Gravato-Nobre et al., 2016). In healthy *C. elegans*, intestinal *ilys-3* expression undergoes a post-developmental regulatory oscillation: levels increase after L1 hatching, decline after the L2 transition, and increase again after L4 transition becoming abundantly expressed in the intestine of adult worms (Gravato-Nobre et al., 2016). Notably, coelomocytes of *C. elegans* also express *ilys-3* (Gravato-Nobre et al., 2016) and are scavenger cells that endocytose fluid from the pseudocoelom (Fares and Greenwald, 2001). The role of coelomocytes in parasitic nematodes is not well understood and they have not been studied in immunity. Given the demonstrated importance of lysozymes for *C. elegans* and the wide distribution of this protein family across species, it is expected that they support helminth survival amongst the host-microbiota.

### Nemapores

Known as caenopores in *C. elegans*, nemapores contain a saposin domain and share similarity with protozoan amoebapores and mammalian NK-lysin and granulysin (Leippe, 1995; Roeder et al., 2010). As with ABFs, nemapores are also cysteine-rich, but contain multiple α-helices (Mysliwy et al., 2010). Nemapores are widely distributed and in addition to *C. elegans*, have been found in 46 nematode species including Ascarids, Trichurids, and the human filarial worms *B. malayi* and *W. bancrofti* (Tarr, 2012). Only caenopores have been studied experimentally, with caenopore-1 and 5 demonstrating antimicrobial activity (Bányai and Patthy, 1998; Roeder et al., 2010). Caenopore-5 is constitutively expressed in the intestine, while caenopore-3 is induced by starvation as well as the bacterial strains *B. megaterium* and *Micrococcus luteus*, suggesting functions in nutrition and immune defense (Roeder et al., 2010). Interestingly, the free-living soil bacteria *B. megaterium* and *M. luteus* are both frequently found with Rhabditid nematodes (Coolon et al., 2009) and may be present in the environment of *C. elegans*. As with lysozymes, experimental evidence of nemapore function in parasitic nematodes is currently lacking.

### Antimicrobial activities of helminth-derived products

In addition to studies demonstrating antimicrobial activities of specific helminth-derived molecules described here, activity can also be detected from the ESPs of parasites. In one study, ESPs from the filarial worm *O. ochengi* were analyzed revealing 36 candidate AMPs (Eberle et al., 2015). Of the 36 candidates, the investigators were able to unambiguously attribute bactericidal activity against *E. coli* to three peptides while the other 33 peptides had been assessed as part of peptide mixtures. Antibacterial activity has also been shown for ESPs from adult *T. suis* worms (Abner et al., 2001). Different bacterial strains were tested, including *S. aureus, E. coli*, and *C. jejuni*. The observed activity was shown to be due to small (<10 kDa), boiling-resistant molecules, indicative of AMPs. Interestingly, pore-forming proteins have also been isolated from *T. trichiura* and *T. muris*, indicating that antimicrobial activity of nematode ESPs is likely due to multiple components of varying sizes and mechanisms (Drake et al., 1994). As nematodes produce a variety of antimicrobial factors, innate immune responses to microbes by helminths likely offer protection using several different strategies.

## Conclusions and perspectives

**Figure 2:**
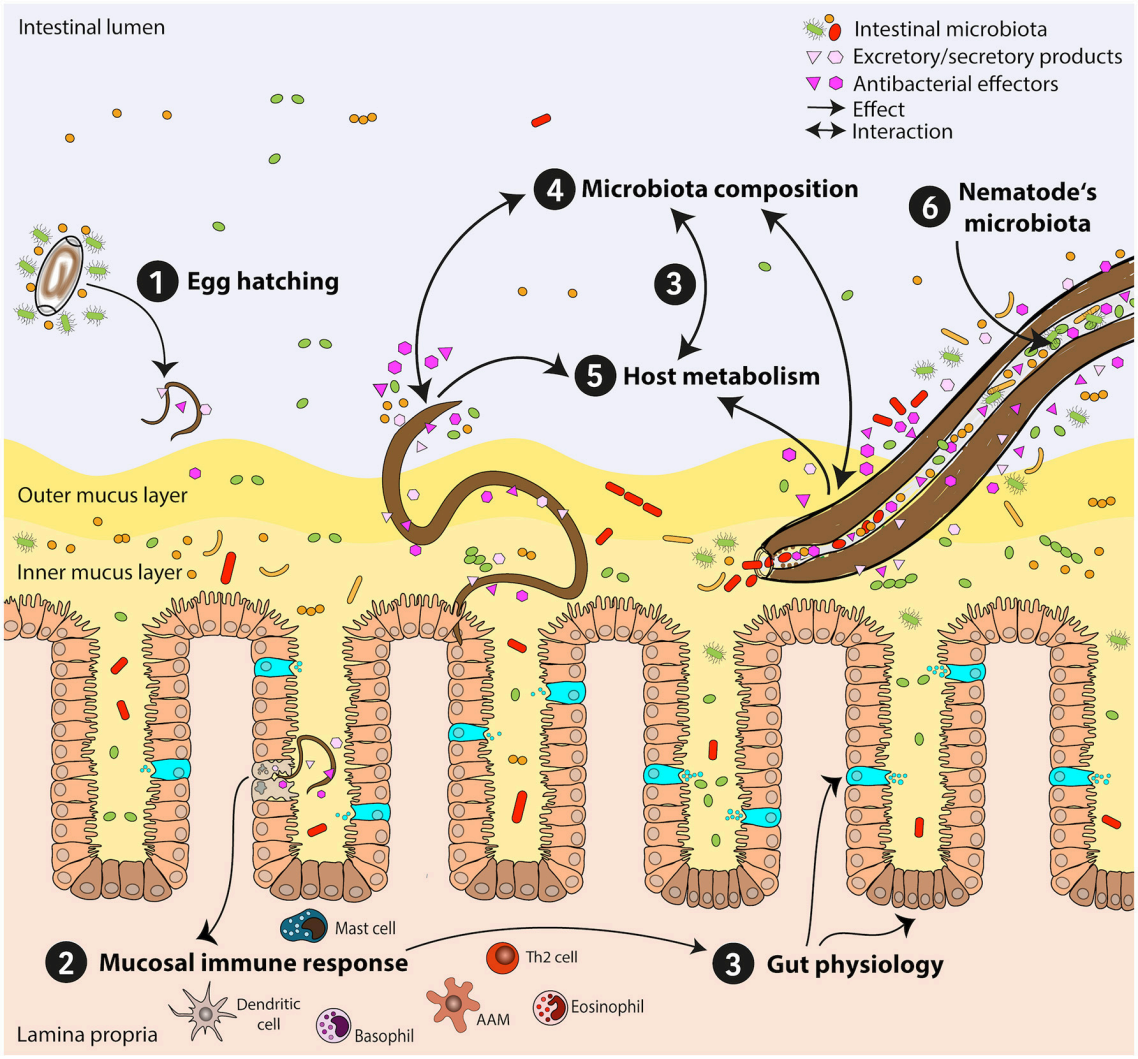
**Mutual influences of intestinal nematodes and host-gut bacteria**. Establishment and persistence of intestinal nematodes in the host's gut are affected by bacterial communities and lead to substantial changes of the gut microbiota. Here, nematode-microbiota interactions and their impact on the host immune response and physiology are exemplified. (1) Egg hatching: Interaction of eggs with the intestinal microbiota is needed for some species, enabling larvae to hatch. (2) Mucosal immune response: Attachment to the epithelium and tissue migratory phase might lead to bacterial translocation and manipulation of immune responses. The anti-helminth immune response is predominantly a T helper type 2 response. (3) Gut physiology: Immune responses induce changes in gut physiology via induction of goblet cell hyperplasia, mucus production, and epithelial turnover, leading to changes in the host microbiota and its metabolome. Specific subsets of bacteria directly influence host physiology through their metabolic activities (e.g., short chain fatty acids-producing bacteria). (4) Microbiota composition: Intestinal nematodes modify intestinal microbial communities via different mechanisms: a) directly via secretion of antibacterial molecules and/or excretory/secretory products, b) indirectly by metabolic and physiologic shifts influencing the gut milieu. Chronic infections often lead to reduction in bacterial diversity and outgrowth of specific bacterial species beneficial for parasite survival. **(5) Host metabolism:** Intestinal nematodes modify host metabolism and nutrient uptake, e.g., alter amino acid, fatty acid, and carbohydrate metabolism, with subsequent influence on gut physiology, immune reactivity, and intestinal microbiota composition. (6) Nematode's microbiota: Nematode-associated bacterial communities might reflect the host microbiota and may also serve as transmission vehicles for pathogenic bacteria. Th2 cell, T helper type 2 cell; AAM, alternatively activated macrophage.

Antibiotic resistance is a rapidly escalating problem with devastating consequences for human and animal health. Drug resistance mechanisms described for microbes and helminths foreshadow the emergence of immense clinical and economic challenges associated with previously treatable infectious diseases. Meanwhile, inflammatory and metabolic diseases also inflict great suffering and are a source of considerable strain on healthcare systems; therefore, novel solutions are sought after to combat these difficulties. As nematodes have evolved over millions of years in a diverse microbial environment, a better understanding of how nematodes interact with microbial populations may offer innovative strategies for treating human and animal diseases. Given the importance of the microbiota and the ability of helminths to influence microbial communities in the gut (Figure 2), helminth infections and helminth products are being studied for their role in dysbiosis. Preliminary data suggest potential benefits of helminths in inflammatory diseases and in individuals with metabolic abnormalities. An additional application of the impact of nematodes on microbes is the development of new antimicrobials. AMPs are under investigation for their therapeutic potential and an understanding of how antimicrobial effectors are used by organisms living amongst diverse microbial populations can guide development efforts. In elucidating the impacts of different bacteria on nematodes, one can anticipate the discovery of novel anthelmintic targets. Moreover, these studies also provide insights into conserved innate immune mechanisms. The divergent environments and experimental systems of parasitic and free-living nematodes offer unique tools to investigators, the combination of which enhance our comprehension of nematode biology and evolutionary ecology.

## Author contributions

All authors listed have made substantial contributions to the planning and writing of this work. All authors have approved this work for publication.

## Funding

The work was supported by the German Research Foundation: GRK 2046 (AM and SH) and HA2542/3-2 (SH).

### Conflict of interest statement

The authors declare that the research was conducted in the absence of any commercial or financial relationships that could be construed as a potential conflict of interest.
